# Spontaneous rupture of solid pseudopapillary tumor of pancreas: A case report and review of literature

**DOI:** 10.1097/MD.0000000000017554

**Published:** 2019-11-01

**Authors:** Xiaofeng Xu, Diyu Chen, Linping Cao, Xiaode Feng, Rongliang Tong, Shusen Zheng, Jian Wu

**Affiliations:** a Division of Hepatobiliary and Pancreatic Surgery, Department of Surgery, First Affiliated Hospital, School of Medicine, Zhejiang University,; b Key Laboratory of Combined Multi-organ Transplantation, Ministry of Public Health,; c Collaborative Innovation Center for Diagnosis Treatment of Infectious Diseases, Hangzhou, Zhejiang, China.

**Keywords:** cyst, neoplasm, pancreas, rupture, solid pseudopapillary tumors

## Abstract

**Introduction:**

Solid pseudopapillary tumors (SPT) account for 1% to 3% of all pancreatic tumors. They have low malignant potential with a favorable prognosis, and predominantly occur in young women. The pathogenesis and clinical behavior of SPT are still uncertain. In addition, most ruptures of SPT were associated with blunt abdominal trauma, while spontaneous ruptures seemed to be quite rare. Up to now, there have been only 3 spontaneous ruptured SPT cases reported worldwide.

**Patient concerns:**

Here, we reported a 22-year-old female patient with left lower abdominal pain. Computed tomography (CT) showed that a hemorrhagic complex solid cystic mass located in the lesser omentum sac.

**Diagnosis:**

According to pathological findings of tumor specimen, the diagnosis of solid pseudopapillary tumor (SPT) of the pancreas was made.

**Interventions:**

Distal pancreatectomy and splenectomy was carried out.

**Outcomes:**

The patient recovered to normal status within 10 days after surgery.

**Conclusion:**

Besides, we reviewed about 50 cases in literatures to find out the clinical characteristics and differential diagnostic strategies of SPT.

## 1 Introduction

Solid pseudopapillary tumor (SPT) of the pancreas is a kind of rare neoplasm, which represents less than 3% of all exocrine pancreatic tumors. SPT is prevalent among young females, with a median age of 20 to 30 years old.^[1,2]^ When SPT represents in male, it has greater malignant potential with a worse prognosis. Besides, most of the ruptures of SPT were associated with blunt abdominal trauma, while the spontaneous ruptures seemed to be quite rare. Furthermore, the symptoms of SPT are not typical in general. Symptoms can occasionally occur due to the size and location of the tumor but usually are nonspecific.^[3]^ Because of its unusual behavior, SPT is often associated with diagnostic and therapeutic challenges.^[4]^ Computed tomography (CT) and magnetic resonance imaging (MRI) is beneficial for the diagnosis of this tumor.^[5]^ Surgical resection is now considered as the most efficient treatment option for patients with SPT, because it offers a good chance of long-term survival.

## 2 Case report

**Figure 1:**
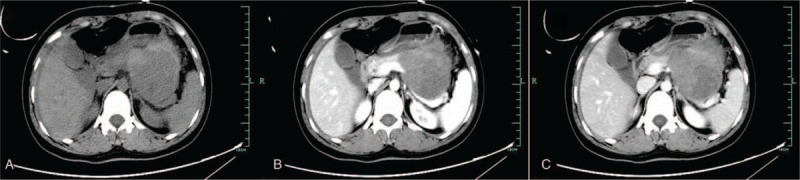
The CT scan demonstrated a cystic lesion (about 95 × 75 mm) located in the pancreas.

The patient was a 22-year-old female, who presented with 2 days history of left lower abdominal pain. She was taken to the local hospital, and the computed tomography scan revealed a large occupying lesion in the peripancreatic clearance. She was treated with antibiotics and intravenous fluid therapy, but the symptom still advanced. Then she was admitted to the emergency department of our hospital. Physical examination revealed the tenderness and rebound-tenderness of the whole abdomen and abdominal muscular tension was obvious. Laboratory findings revealed elevated leukocytosis (12.3 × 10^9^/L), neutrophile granulocytes (86.3% of the leukocytes) and decreased hemoglobin (Hb) (105 g/L). Then a CT scan was performed again to assess the properties of the abdominal lesion. The review result of the CT scan showed a hemorrhagic complex solid cystic mass located in the lesser omentum sac, which was considered to be originated from pancreas (Fig. 1).

**Figure 2:**
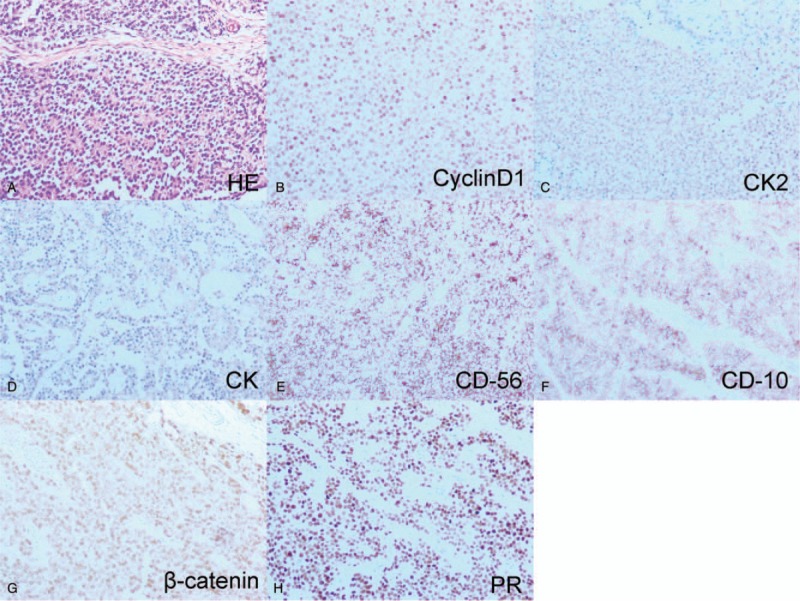
A. H&E staining shows that gland-like structure was lined by round tumor cells in solid pseudopapillary tumors specimen (original magnification × 100). B–H: The immunohistochemical results of solid pseudopapillary tumors specimen.

**Figure 3:**
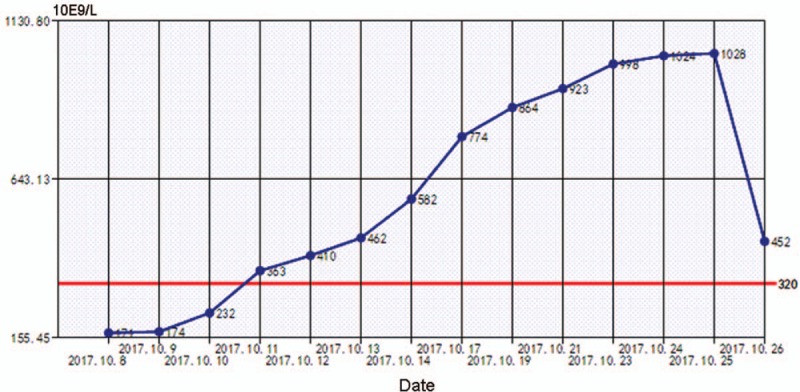
Trend of platelet change after surgery of this patient.

**Figure 4:**
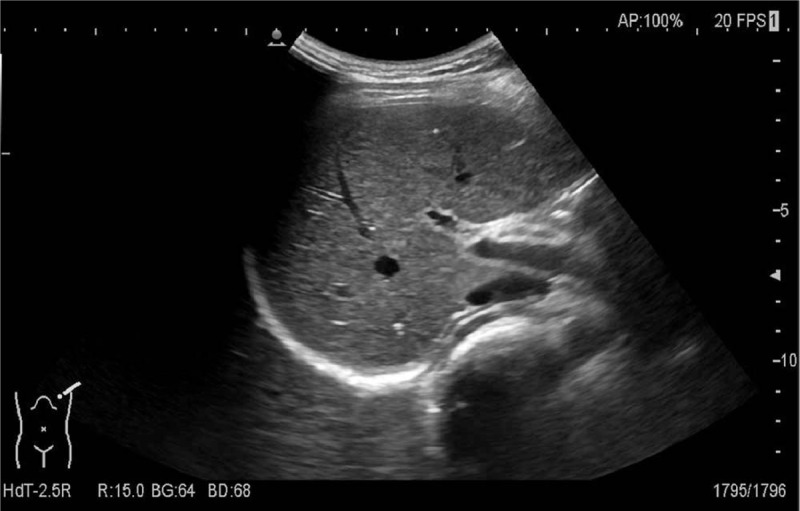
Abdominal ultrasound scan at 10 months after operation.

In order to stop bleeding in time, the patient underwent emergency excision laparotomy of the cyst based on clinical and radiological findings. We located the tumor in the body of the pancreas (about 8 × 7 cm), and it had invaded to the spleen. Therefore, distal pancreatectomy (including the cyst, the body and tail of pancreas) and splenectomy were performed. The pathology results reported a solid-cystic mass (8 × 6.5 × 5 cm) with heterogenesis. Histological examination indicated that a solid and vascular pattern with pseudo-papillary cores (Fig. 2). Immunohistochemical (IHC) stains for CK (pan), CD-10, PR, CyclinD1, CD-56 and β-Catenin showed positivity, but CgA and E-cadherin staining were negative. On the whole, final pathological diagnosis was solid pseudopapillary tumor of the pancreas. In the postoperative period, the patient had high level of platelet (PLT) (12.3 × 10^9^/L, Fig. 3), and it was thought to be induced by the resection of spleen. After treated by acute preoperative plateletpheresis, PLT level came back to normal. A follow-up 10 months later showed neither signs of tumor recurrence nor endocrine and exocrine insufficiency of the pancreas (Fig. 4).

## 3 Discussion

### 3.1 Description of solid pseudopapillary tumors of pancreas

SPT is an uncommon and enigmatic pancreatic neoplasm firstly described by Frantz in 1959,^[3]^ which is considered to be low malignant potential.^[6]^ It represents 0.2% to 2.7% of tumors in the pancreas.^[7]^ Frequently, it is identified as solid and cystic tumor, solid and papillary epithelial neoplasm, papillary-cystic neoplasm, papillary-cystic epithelial neoplasm, papillary-cystic tumor or Franz tumor. The concept of SPT for the international histological classification of tumors of the exocrine pancreas was firstly put forward by the World Health Organization (WHO) in 1996.^[8]^ Then we collected 50 SPT cases from pubmed database to ensure the characters of SPT. Until now, the cases reported showed that SPT commonly occurs in the head or tail region of pancreas. As shown in Table 1, 17 of the cases had the SPT in the head of pancreas. While, 5 reported cases had SPT in the body, and 8 cases occurred in the tail. The remaining cases reported the tumor located in the head-body junction (4/50) or, in the body-tail junction of pancreas (10/50).

**Table 1:**
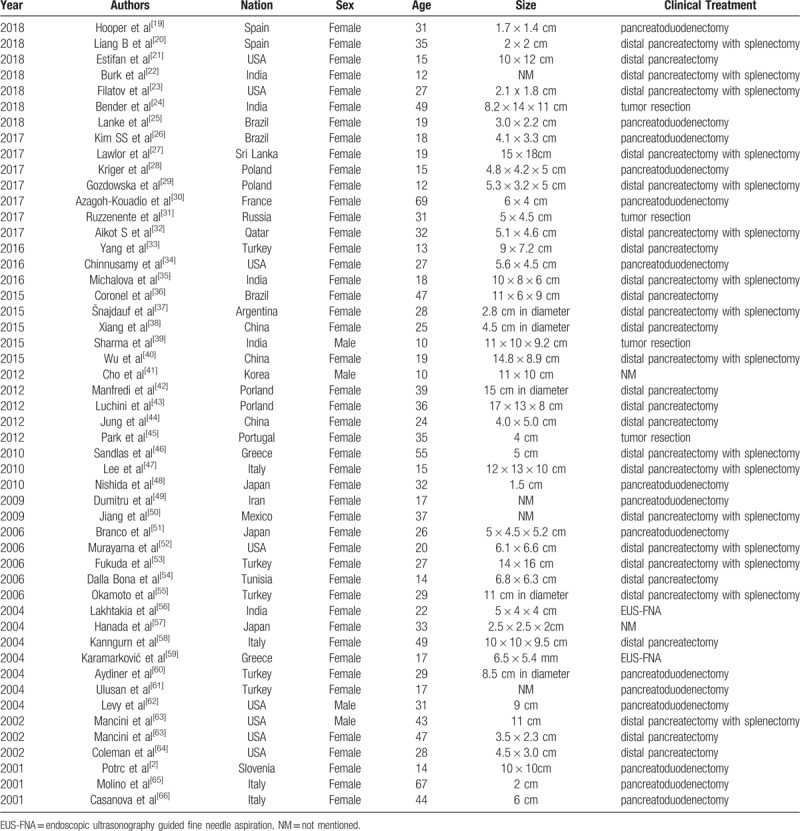
Summary information of the 50 cases reviewed in literatures.

### 3.2 Spontaneous rupture of solid pseudopapillary tumors of pancreas

The symptoms of SPT are usually nonspecific, with abdominal pain being the most common. SPT which was discovered after rupture and hemoperitoneum was rare.^[9]^ According to the cases reported, most ruptured SPTs are induced by the blunt abdominal trauma, and spontaneous ruptures seem to be quite uncommon. Since the cystic part of SPT consisted of the degeneration after the intramural hemorrhage, SPT had a natural tendency to hemorrhage inside the tumor.^[10]^ Abrupt massive hemorrhage and increased pressure of the tumor are considered to be the main reasons for the spontaneous rupture of SPT. When we face with such patient, the enhanced CT and emergency laparotomy would be helpful to make a correct diagnosis.

### 3.3 Clinical findings of solid pseudopapillary tumors of pancreas

We reviewed 50 case reports of SPT in literatures. The patients included 46 females and 4 males, the ages ranged from 10 to 69, and the mean age was 31.5 years old (Table 1). Totally, SPT, as an uncommon, typically benign tumor, is found mainly in young non-Caucasian women between the 2nd and 3rd decades of life. In addition, we found that most of the patients were Asians (20/50) and Americans (18/50), and Europeans comprised 11/50, respectively (Table 2). This observation suggests that it seems to have a predilection for Asian and American women, although rare cases have been reported in children and men. Some experts considered that female predominance may attribute to the proximity of primordial pancreatic cells to the ovarian ridge during the development of SPT.

**Table 2:**
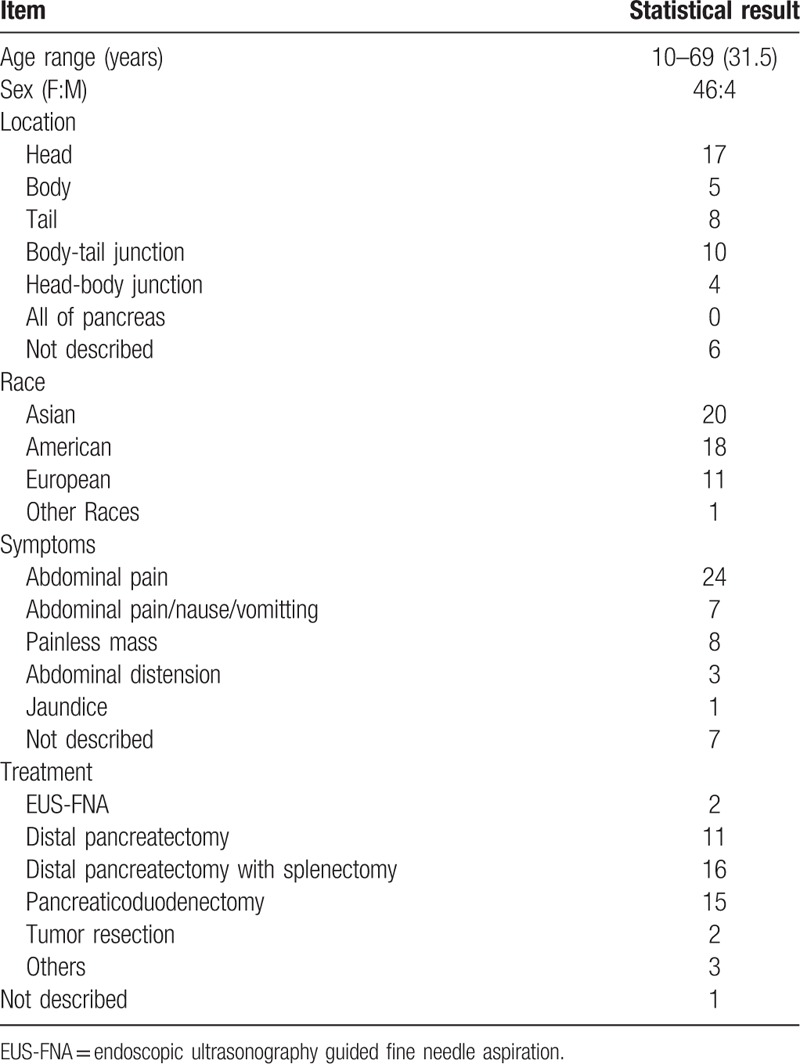
Epidemiological and pathological results of the 50 cases in the literature review.

Clinical presentation of solid pseudopapillary tumor in pancreas is various. Abdominal discomfort or vague pain can be the most common symptom, but the minority of patients can be also asymptomatic and the tumors are detected incidentally. However, some symptoms including vomiting, discomfort in the epigastrium or jaundice occur more rarely.^[11]^ We collected about 50 cases of SPT between 2018 to 2001 (Table 1). As the reported cases showed (Table 2), most patients presented with abdominal pain (24/50), and others complained with the symptoms of nausea, vomiting (7/50), abdominal distension (3/50), or jaundice (1/50). However, when the tumors invade to neighboring organs, such as the adrenal glands, patients present with specific clinical manifestations, such as acute kidney injury (AKI) with rhabdomyolysis. Therefore, the clinical presentations may be the necessary clues to find the origin of the tumor and make the differential diagnosis.

### 3.4 Diagnosis of solid pseudopapillary tumors of pancreas

Image examination is significant in diagnosis of SPT. On X-ray, solid pseudopapillary tumors appear as large masses, which sometimes could displace adjacent structures like stomach or bowels. The majority of tumors are diagnosed through ultrasound or CT scan of the abdomen. Ultrasound shows a well-defined mass with solid and cystic components and increased vascularity.^[12,13]^ Besides, CT imaging is superior for the diagnosis of SPT. Through contrast enhanced CT, it shows an encapsulated lesion with enhancing solid and non-enhancing cystic areas with some showing calcific foci. If spontaneous bleeding was occurred in the tumor, the hemorrhagic density can be found within the lesion. Solid pseudopapillary tumors may grow to large sizes with a mean diameter ranging from 6 cm to 10 cm.^[14]^ Through magnetic resonance image (MRI), it reveals the hyper-vascular, well-encapsulated, round tumors with mixed cystic and solid components. Furthermore, echo-endosonography may provide FNA biopsy with the possibility of pre-operative pathologic diagnosis. So SPT requires differential diagnosis to be made with other pancreatic tumors, such as mucinous neoplasm, serous cystadenoma, pseudocyst, nonhyperfunctioning islet cell tumor and pancreatic adenocarcinoma (Table 3). Thus, to ensure a better surgical approach, a clear preoperative diagnosis of SPT is preferable.

**Table 3:**
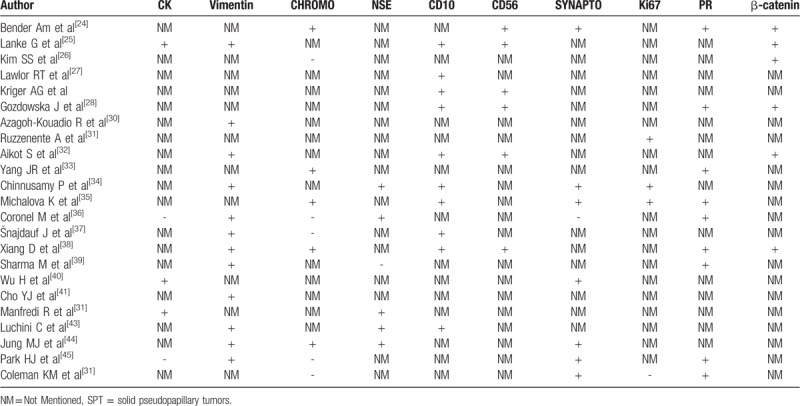
Immunohistochemical markers of the SPT specimens in the literature review.

As for the confusion between pancreatic SPTs and cystic neoplasms, histological differential diagnosis is crucial. SPT is composed of poorly cohesive, monomorphic cells forming solid, and pseudopapillary structures are lined by neoplastic cells. Moreover, cystic spaces containing blood and necrotic debris.^[15]^ Histologically, the neoplastic cells are characteristically strongly positive for vimentin, α1-antitrypsin, α1-antichymotrypsin, CD-10, progesteron receptor (PR), neuronspecific enolase, CD-56 and cyclin D1.^[6,16]^ In our review, we found that neoplastic cells were strongly and diffusely positive for vimentin in 12 cases, positive for CD-10 in 10 cases. Besides, a total of 9 patients in our review showed positive results for PR expression and 8 patients showed positive results for α1-antitrypsin expression (Table 4). Thus, the combination of multiple IHC markers including vimentin, CD-10, PR andα1-antitrypsin, may contribute to improving the diagnosis rate of SPT.

**Table 4:**
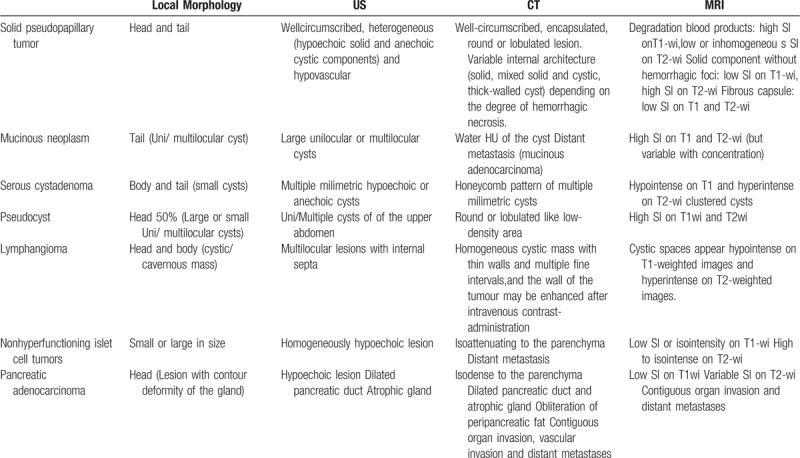
Differential diagnosis of SPT.

### 3.5 Treatments for solid pseudopapillary tumors of pancreas

Despite the large tumor size at the time of diagnosis, surgery is the preferred treatment option for solid pseudopapillary tumors of pancreas. In addition, complete aggressive resection is necessary for SPT. Based on the morphology and size of the tumor, different surgical options, including a simple excision of the mass or a pancreatic resection, such as pancreaticoduodenectomy or distal pancreatectomy, must be considered.^[17]^ Due to the potential malignancy, liver is the most common metastatic site of SPT. For liver metastases, other treatments include chemotherapy, alcohol injection, transcatheter arterial chemoembolization, radiotherapy, and liver transplantation could be considered.^[18]^ However, distant or local recurrences still could occur in some cases after surgical resection. The recent studies showed that moreover repeated surgical resection for recurrences can considerable prolong survival. This case was admitted to our hospital for acute hemorrhage, emergency surgery was performed. However, as SPT is a potential benign and malignant borderline tumor, the choice of emergency surgery may cause high risk for recurrence of the patient. Therefore, when it is possible to stabilize the condition of patient with conservative treatment, elective surgery may be a better choice.

## Author contributions

**Investigation:** Linping Cao, Xiaode Feng.

**Methodology:** Linping Cao.

**Resources:** Diyu Chen.

**Writing – original draft:** Xiaofeng Xu, Diyu Chen, Xiaode Feng, Jian Wu.

**Writing – review & editing:** Rongliang Tong, Shusen Zheng, Jian Wu.
